# Incidence of Childhood Dilated Cardiomyopathy in Sweden From 1991 to 2019: A National Population‐Based Study

**DOI:** 10.1111/apa.70672

**Published:** 2026-07-02

**Authors:** Shalan Fadl, Stefan Särnblad, Tahir Taj, Håkan Wåhlander, Eva Fernlund, Jan Sunnegårdh

**Affiliations:** ^1^ Department of Paediatrics, Faculty of Medicine and Health Örebro University Örebro Sweden; ^2^ Department of Obstetrics and Neonatology Capio St Görans Hospital Stockholm Sweden; ^3^ Clinical Epidemiology and Biostatistics Unit Örebro University Hospital Örebro Sweden; ^4^ Department of Paediatrics, Institute of Clinical Sciences University of Gothenburg Gothenburg Sweden; ^5^ Paediatric Heart Centre, Queen Silvia Children's Hospital Sahlgrenska University Hospital Gothenburg Sweden; ^6^ Crown Princess Victoria Children's Hospital, and Paediatric Clinic, Vrinnevi Hospital, Department of Biomedical and Clinical Sciences and Department of Paediatrics Linköping University Linköping Sweden

**Keywords:** cohort study, congestive heart failure, heart transplantation, mortality, outcome

## Abstract

**Aim:**

To study the incidence, survival, and mortality of dilated cardiomyopathy (DCM) in children in Sweden, 1991–2019.

**Methods:**

Hospital records of 0–18‐year‐olds diagnosed with DCM over a 29‐year period were reviewed. The annual sex‐specific incidence rates of DCM were calculated as new disease cases, divided by the average Swedish same‐sex population for each year between 1991 and 2019.

**Results:**

Altogether 377 patients were identified, 204 (54%) male and 173 (46%) female. The annual incidence rate of DCM over the study period was 0.62/100 000 person‐years. The incidence rate was 5.98/100 000 persons in patients < 1 year old compared with 0.33/100 000 in 1–18‐year‐olds. Linear regression analysis revealed no change (*p* = 0.82) in the incidence of DCM over the 29‐year period. Among children with a clinical diagnosis and available follow‐up (*n* = 268), the mortality rate was 23%. Children aged > 10 years at diagnosis experienced an increased risk of death compared with children ≤ 3 months of age at diagnosis (hazard ratio 1.92). The heart transplant rate was 26%.

**Conclusions:**

In this national study, we found an incidence rate of paediatric DCM of 0.62/100 000 person‐years in Sweden. The mortality risk was highest in children > 10 years old at diagnosis.

## Highlights

- Insights into the incidence and outcomes of childhood dilated cardiomyopathy at the national level in Sweden are lacking.
- We observed an incidence of dilated cardiomyopathy of 0.62/100 000 person‐years in Sweden.
- The recovery of left ventricular function is promising, with 70% of living, non‐transplanted patients regaining normal heart function.

**Abbreviations**
AV: atrioventricular
CDR: Cause of Death Register
CHF: congestive heart failure
CI: confidence interval
DCM: dilated cardiomyopathy
ECHO: echocardiogram
ECMO: extracorporeal membrane oxygenation
FDCM: familial dilated cardiomyopathy
FS: fractional shortening
HR: hazard ratio
ICD‐9: International Classification of Diseases, 9th revision
ICD‐10: International Classification of Diseases, 10th revision
ICD: International Classification of Diseases
ICU: intensive care unit
IDCM: idiopathic dilated cardiomyopathy
LVEDd: left ventricular end‐diastolic diameter
LVESd: left ventricular end‐systolic diameter
LVFS: left ventricular fractional shortening
LVNC: left ventricular non‐compaction
NPR: National Patient Register
SD: standard deviation
SDS: standard deviation score
VDCM: post‐viral dilated cardiomyopathy
WHO: World Health Organization

## 1 Introduction

Childhood dilated cardiomyopathy (DCM) is an uncommon disease characterised by dilation and impaired function of the left or both ventricles [1, 2]. Dilated cardiomyopathy usually presents as congestive heart failure (CHF) or arrhythmia. As the leading diagnosis requiring cardiac transplantations in children and adolescents, DCM accounts for > 50% of heart transplantations [3, 4, 5] and is associated with significant morbidity and mortality. Advances in genetic techniques have increased the knowledge about the multifactorial aetiologies of this disorder; still, over half of cases today are classified as idiopathic [1, 6, 7, 8]. We previously reported a combined incidence of DCM and LVNC of 0.77/100 000 person‐years in a regional Swedish cohort [9]. In that study, 30% of children died or received a heart transplantation within 1 year after diagnosis. Another Swedish study reported that 12.2% of 15–35‐year‐olds who died from sudden cardiac death were diagnosed with DCM at autopsy [10]. There are no Swedish national data on the occurrence and outcomes of childhood DCM.

### 1.1 Aim

This study aimed to investigate the incidence of and outcomes in childhood DCM in a national cohort during 1991–2019.

## 2 Patients and Methods

### 2.1 Setting and Participants

Eligible patients were identified from the National Patient Register (NPR). Since 1987, the NPR has included all inpatient care in Sweden, and since 2001, it has also covered outpatient visits in specialised care [11]. Data were also collected from the Swedish Cause of Death Register (CDR) to identify deceased individuals. The NPR and CDR are held by the National Board of Health and Welfare in Sweden. Data for this study were obtained for the period 1991–2019.

Patients were identified using International Classification of Diseases (ICD), 9th revision (ICD‐9), code 42.5E and 10th revision (ICD‐10) codes I42.0, I42.7, I42.8, and I42.9 in the NPR or CDR.

### 2.2 Inclusion and Exclusion Criteria

Subjects aged 0–18 years who were diagnosed with DCM between 1 January 1991 and 31 December 2019 were eligible for this study. The diagnosis recorded in the NPR or CDR was first verified in the medical records. Only subjects with a valid ICD code who met the World Health Organization (WHO) criteria were included in further data collection. The 1995 World Health Organization/International Society and Federation of Cardiology Task Force defined dilated cardiomyopathy as dilation and impaired contraction of the left ventricle or both ventricles [12]. Specifically, this condition is defined as the dimension of the left ventricle being ≥ 2 SDs above the normal mean, and/or the left ventricular fractional shortening (LVFS) below the normal range [13]. A fractional shortening (FS) threshold of ≤ 27% that had been confirmed by echocardiography was employed as the diagnostic criterion in clinically diagnosed cases, and the disease was verified for ≥ 3 months.

In instances of post‐mortem cases involving unexpected deaths, where there had been no prior contact with a paediatric or cardiology unit, inclusion was permitted if the ICD‐9/10 code indicated DCM and no alternative aetiology was identified. This inclusion criterion applied even in the absence of echocardiographic evidence or compliance with the duration criteria of three months.

Children with asymptomatic FDCM diagnosed during family genetic or clinical screening between January 1991 and December 2019 were excluded. Subjects with LVNC not fulfilling other inclusion criteria for DCM were excluded as the European Task Force does not consider LVNC to be a cardiomyopathy in the general sense [14, 15, 16, 17, 18]. Subjects with cardiac disease due to structural heart disease, muscular dystrophy, chronic arrhythmia, grade 3 atrioventricular (AV) block, or pacemaker‐induced DCM were excluded as the pathophysiology of these disorders is different. Subjects with arrhythmogenic right ventricular cardiomyopathy as the main diagnosis were likewise excluded.

### 2.3 Data Collection From Medical Records

Data collection for identified patients was performed from medical records. Complete medical records of all patients except those with DCM diagnosed post‐mortem were retrieved from hospitals across Sweden (with the exception of one paediatric unit that did not send records for ten patients). These hospitals served about 8.6 million people in 1991 and 10.3 million in 2019. A list of post‐mortem‐diagnosed DCM cases was retrieved from the CDR.

Subsequently, one investigator (SF) extracted data from medical records from diagnosis until 31 December 2019. The data collection was performed from 2017 to 2022.

Data comprised medical history including CHF symptoms and arrhythmia, history of respiratory and other infections up to 3 months before diagnosis, family history of cardiac disease documented at diagnosis, and age and sex. The date of initial presentation was defined as the first symptomatic presentation and the first record of left ventricular dilation and dysfunction.

We documented the clinical conditions and investigations at diagnosis and after 3, 6 and 12 months and then at 12‐month intervals until the last follow‐up visit.

Echocardiographic M‐mode measurements of left ventricular end‐diastolic diameter (LVEDd) and left ventricular end‐systolic diameter (LVESd) were used to calculate body surface area‐adjusted Z‐scores, based on length and weight, following the Detroit Z‐score method [19]. Data on transplantation and/or mortality were obtained from medical records, NPR and the CDR.

### 2.4 Definitions of Subgroups

Idiopathic dilated cardiomyopathy (IDCM) was defined as a lack of family history and no known infectious or metabolic causes. Familial DCM (FDCM) was considered if there was a first‐degree relative with primary DCM and the condition was identified through clinical screening or genetic testing. Post‐viral dilated cardiomyopathy (VDCM) was considered if the disease had developed after viral myocarditis with/without positive viral serology.

If the patient had an inborn error of metabolism, or nutritional or endocrine causes, the disease was considered to be metabolic. Metabolic cases, along with cases caused by cardiotoxic drugs, are collectively referred to as “other” or “other‐cause DCM”.

### 2.5 Statistical Analysis

The annual sex‐specific incidence rates of DCM were calculated as new disease cases, divided by the average Swedish same‐sex population for each year between 1991 and 2019.

Post‐mortem‐diagnosed DCM (*n* = 93) were excluded from the clinical management analysis of the patients with CHF at diagnosis. Patients with incomplete follow‐up data (*n* = 16) and 93 individuals diagnosed post‐mortem with DCM of unknown aetiology were excluded from the analysis of patient outcome based on aetiology. Descriptive statistics were used to summarise patient characteristics, with categorical variables presented as counts and percentages, and continuous variables as mean, SD and standard deviation score (SDS). For the survival analysis, we applied the Kaplan–Meier method to estimate the proportion surviving and 95% confidence intervals (CIs) at 3 and 6 months, and 1, 2, 3, 5, 7, 10, 15 and 18 years post‐diagnosis. Transplantation was treated as a censoring event, and the endpoint for Cox regression was all‐cause mortality. The endpoint was defined as death (event) or censoring at transplantation or the last follow‐up visit to a paediatric cardiologist, whichever occurred first. Statistical analyses were performed using R statistical software (R Foundation for Statistical Computing, Vienna, Austria).

### 2.6 Ethical Standards

The research adhered to the ethical principles outlined in the 1975 Declaration of Helsinki. The study protocol received approval from the Joint Commission on Ethics at Sahlgrenska University in Gothenburg, Sweden, on 12 January 2017 (ref. No. Dnr 857–16). Additionally, ethical modifications pertaining to the study were approved on 2 July 2020 (ref. No. Dnr 2019–05647), covering the period from 2017 to 2020.

## 3 Results

### 3.1 Incidence

A total of 377 patients with DCM were identified from the NPR and CDR, which included 93 (25%) cases diagnosed post‐mortem (Figure 1). Of these patients, 204 (54%) were male and 173 (46%) female. The distribution of age at diagnosis was skewed, with a mean (±SD) of 2.5 ± 3.6 years, and median (min–max) of 3.0 (0–18) years. The age distribution at diagnosis revealed that 99 children (26%) were diagnosed at < 3 months of age, 88 (23%) between 3 months and 1 year, another 83 (22%) between > 1 and 10 years, and 107 (29%) between > 10 and 18 years. The follow‐up duration for these patients averaged 7.7 years, with a median of 5.0 (range 0–18) years. A small numerical male predominance was noted in most age groups. The clinical characteristics of patients across all groups are detailed in Table 1. The overall incidence rate of DCM in Sweden from 1991 to 2019 was calculated to be 0.62/100 000 person‐years (95% CI 0.56–0.69). The incidence was 0.65/100 000 person‐years among males and 0.59/100 000 person‐years among females. This difference was not statistically significant, as illustrated in Table 2. Notably, the annual incidence rate for children aged < 1 year was higher, at 5.98 cases per 100 000 persons (95% CI 5.15–6.90), while the rate for those aged 1–18 years was 0.33 cases per 100 000 persons (95% CI 0.28–0.38). The linear regression analysis (Figure 2) revealed no change (*p* = 0.82) in the incidence of DCM over the 29‐year period.

**FIGURE 1:**
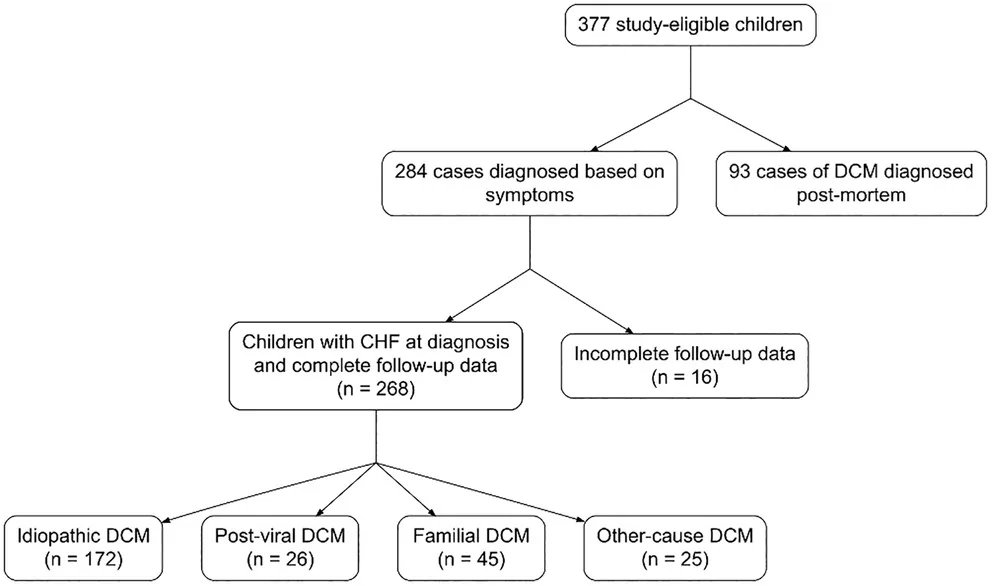
Children included in the study. CHF = congestive heart failure; DCM = dilated cardiomyopathy.

**TABLE 1:** Patient characteristics by aetiology (*n* = 377).

Patient characteristics	IDCM^a^, *n* (%) 172 (45)	VDCM^b^, *n* (%) 26 (7)	FDCM^c^, *n* (%) 45 (12)	Other^d^, *n* (%) 25 (7)	Unknown causes^e^, *n* (%)109 (29)	All patients, *n* (%) 377 (100)
Sex						
– Male	88 (51)	15 (58)	23 (51)	16 (64)	62 (57)	204 (54)
– Female	84 (49)	11 (42)	22 (49)	9 (36)	47 (43)	173 (46)
Age at diagnosis						
– < 3 months	57 (33)	3 (12)	16 (35)	6 (24)	17 (16)	99 (26)
– 3–12 months	45 (26)	13 (50)	9 (20)	3 (12)	18 (16)	88 (23)
– > 1–10 years	22 (13)	5 (19)	12 (27)	7 (28)	37 (34)	83 (22)
– > 10–18 years	48 (28)	5 (19)	8 (18)	9 (36)	37 (34)	107 (29)
LVEDd^f^ at diagnosis (*n* = 268), cm, mean (SD)	4.5 (1.6)	5.0 (1.4)	4.3 (1.4)	4.4 (1.4)	—	4.5 (1.4)
LVEDd, SDS^g^ at diagnosis (*n* = 243), mean (SD)	4.6 (2.1)	5.8 (2.7)	4.0 (2.2)	3.2 (2.0)	—	4.4 (2.2)
LVFS^h^ at diagnosis (*n* = 273), %, mean (SD)	15 (6.6)	13.3 (6.1)	16.6 (7.2)	20.4 (5.1)	17 (7.8)	16.5 (6.6)
Mitral valve regurgitation at diagnosis (*n* = 277)						
– none/negligible	65 (38)	7 (27)	2 (5)	14 (52)	6 (4)	94 (34)
– mild	59 (34)	12 (46)	23 (51)	7 (28)	2 (2)	103 (37)
– moderate	40 (23)	6 (23)	14 (31)	2 (8)	2 (2)	64 (23)
– severe	8 (5)	1 (4)	6 (13)	0	1 (1)	16 (6)

^a^ Idiopathic dilated cardiomyopathy.

^b^ Post‐viral dilated cardiomyopathy.

^c^ Familial dilated cardiomyopathy.

^d^ Other causes: Metabolic, nutritional, endocrine, cardiotoxicity.

^e^ Unknown causes: Subset of patients with incomplete follow‐up data, *n* = 16. Among these 16 patients, certain echo variables, specifically LVFS (*n* = 5) and mitral regurgitation (*n* = 11), were only documented in the clinical records. Additionally, the subset included 93 cases of DCM that were confirmed through post‐mortem diagnoses.

^f^ Left ventricular end‐diastolic diameter.

^g^ Standard deviation score (Detroit Z‐score used).

^h^ Left ventricular fractional shortening.

**TABLE 2:** Incidence of childhood dilated cardiomyopathy overall, by sex, and age, 1991–2019.

Population Group	Number of cases	Overall incidence per 100 000	95% CI
Overall	377	0.625	0.564–0.691
Female	173	0.590	0.505–0.685
Male	204	0.658	0.571–0.755
Age < 1 year	187	5.98	5.15 – 6.90
Age ≥ 1 year	190	0.334	0.288–0.385
Age ≥ 1 year, Female	77	0.277	0.219–0.346
Age ≥ 1 year, Male	113	0.388	0.320–0.466

**FIGURE 2:**
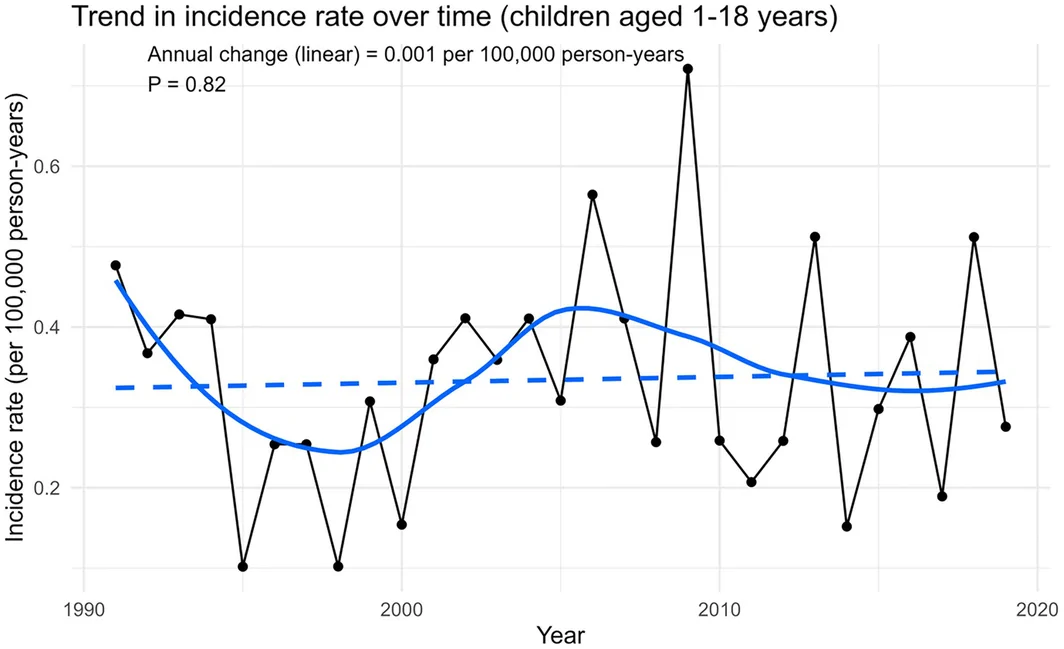
Childhood dilated cardiomyopathy (DCM) incidence trend, 1991–2019. The black line with points represents the yearly observed incidence rates. The smoothed line shows the overall pattern over time. Linear regression showed no significant change in incidence during the study period (*p* = 0.82).

### 3.2 Clinical Symptoms and Management

At the time of diagnosis, 284 patients (75%) were reported to have CHF. Clinical management details are summarised in Table 3. Notably, the 93 patients (25%) diagnosed at post‐mortem were excluded from this analysis. A large number of patients with CHF, 173 patients (60%), were admitted to the paediatric intensive care unit (ICU) for intravenous inotropic support, with 101 patients (35%) requiring mechanical assist devices. A total of 113 patients (40%) were subsequently placed on the transplantation list, as illustrated in Table 3. The need for admission to the ICU was most pronounced among patients with **IDCM;** 137 patients (80%) required ICU admission. Within the IDCM group, 68 patients (39%) were treated with extracorporeal membrane oxygenation (ECMO) or ECMO followed by a left ventricular assist device within the first 6 months post‐diagnosis. Within the IDCM group, 61 patients (35%) were placed on the transplantation list, while 44 patients (26%) ultimately proceeded to transplantation (Tables 3 & 4). Among patients with **VDCM**, 17 patients (65%) were admitted to the ICU for intravenous inotropic support, while ten (38%) received mechanical assist devices (ECMO or ECMO plus assist device) during the initial 6 months post‐diagnosis. Twelve patients (46%) were placed on the heart transplantation list, and five (19%) were transplanted (Tables 3 & 4). Among the **FDCM** group, 15 patients (33%) were admitted to the ICU for intravenous inotropic support; all 15 patients received mechanical support. A total of 33 (73%) of the familial cases were placed on the transplantation list. Only 15 patients (33%) were transplanted (Tables 3 & 4). Most cases of **other‐cause DCM** were attributed to metabolic disorders and nutritional and endocrine causes, along with cases caused by cardiotoxic drug exposure. Three patients (12%) were admitted to the ICU for intravenous inotropic support. Seven patients (28%) received mechanical support; all of them were subsequently listed for transplantation, and all were transplanted, including one patient with drug‐induced cardiotoxicity (Tables 3 & 4).

**TABLE 3:** Clinical management of the patients with congestive heart failure (CHF) at diagnosis (*n* = 284)*.

Patients, *n* (%)	IDCM^a^ 172 (60)	VDCM^b^ 26 (9)	FDCM^c^ 45 (16)	Other^d^ 25 (9)	Unknown aetiology^e^ 16 (6)	All patients 284 (100)
Admission to the paediatrics unit	155 (90)	26 (100)	31 (69)	21 (84)	6 (37)	239 (84)
ICU^f^ > 1 day with inotropic treatment	137 (80)	17 (65)	15 (33)	3 (12)	1 (6)	173 (60)
Mechanical support^g^	68 (39)	10 (38)	15 (33)	7 (28)	1 (6)	101 (35)
Listed for heart transplantation	61 (35)	12 (46)	33 (73)	7 (28)	—	113 (40)
Treatment^h^ (*n* = 229)^i^						
– one medication	9 (5)	2 (7)	8 (18)	0	—	19 (8)
– at least two medications	72 (42)	6 (23)	4 (9)	0	—	82 (36)
– more than two medications and/or anticoagulants	83 (48)	18 (70)	19 (42)	7 (28)	1 (6)	128 (56)

* Excluded individuals: Post‐mortem‐diagnosed DCM (*n* = 93).

^a^ Idiopathic dilated cardiomyopathy.

^b^ Post‐viral dilated cardiomyopathy.

^c^ Familial dilated cardiomyopathy.

^d^ Other causes (nutritional, metabolic, endocrine, cardiotoxicity).

^e^ Patients with incomplete follow‐up data.

^f^ Intensive care unit.

^g^ Extracorporeal membrane oxygenation (ECMO) or ECMO followed by a left ventricular assist device (LV‐device).

^h^ Loop diuretics, mineralocorticoid receptor antagonists, digoxin, angiotensin‐converting enzyme inhibitors, angiotensin receptor blockers, beta‐blockers, I_f_ channel inhibitors, aspirin and other anticoagulants.

^i^ Excluding cases with missing treatment data at diagnosis (*n* = 55), where either the patients did not receive treatment, or the details of the treatments administered were not documented.

**TABLE 4:** Outcomes by aetiology among children with clinical diagnosis and available follow‐up (*n* = 268)*.

Outcome	IDCM^a^ *n* (%)	VDCM^b^ *n* (%)	FDCM^c^ *n* (%)	Other^d^ *n* (%)	All patients *n* (%)
Patients with clinical diagnosis and available follow‐up, *n*	172	26	45	25	268
Transplantation	44 (26)	5 (19)	15 (33)	7 (28)	71 (26)
Death	39 (23)	4 (15)	12 (27)	7 (28)	62 (23)
Alive without transplantation, *n*	89	17	18	11	135
Recovered, LVFS > 27% at last visit	71 (80)	15 (88)	0 (0)	8 (73)	94 (70)
Not recovered, LVFS ≤ 27% at last visit	18 (20)	2 (12)	18 (100)	3 (27)	41 (30)

*Note:* Percentages for transplantation and death are calculated using all children with a clinical diagnosis and available follow‐up within each aetiology group. Percentages for recovered and not recovered are calculated among living, non‐transplanted children.

Abbreviation: LVFS, left ventricular fractional shortening.

* Sixteen patients with incomplete follow‐up data and 93 children with post‐mortem‐diagnosed DCM were not included in the outcome analysis.

^a^ IDCM = idiopathic dilated cardiomyopathy.

^b^ VDCM = post‐viral dilated cardiomyopathy.

^c^ FDCM = familial dilated cardiomyopathy.

^d^ Other = metabolic, nutritional, endocrine or cardiotoxic causes.

### 3.3 Outcome

Outcome data are presented in Table 4. Patients with incomplete follow‐up data (16 individuals) and those diagnosed at post‐mortem (93 individuals) were excluded from this analysis. A total of 62 children (23%) died during the follow‐up period. The mortality rates were observed to be 28% among patients with metabolic and post‐cardiotoxic aetiologies, whereas post‐viral cases exhibited a mortality rate of 15%. This difference in mortality rates was not statistically significant.

Among patients with a clinical diagnosis and available follow‐up (*n* = 268), 113 patients (42%) were placed on the transplantation list, and 71 patients (26%) proceeded to transplantation, as detailed in Table 4. Subsequently, 18 patients died while awaiting transplantation, whereas 24 individuals demonstrated sufficient recovery to be removed from the transplantation list. The heart transplantation rates were 33% in patients with FDCM, 28% in those with other‐cause DCM, 19% in patients with post‐viral DCM, and 26% in patients with IDCM. These differences were not statistically significant.

Recovery of left ventricular function, defined as LVFS > 27%, was documented in several patients. Among the 135 living, non‐transplanted children, 94 (70%) had normal left ventricular function at the latest follow‐up. Recovery was observed in all groups except in the familial cases, as shown in Table 4. Consequently, at the latest follow‐up, 41 (30%) of the 135 living, non‐transplanted children with DCM exhibited sustained reduced left ventricular function. Kaplan–Meier survival analysis indicated 75% survival among children diagnosed at or before 10 years of age, and the highest mortality occurred within the first year after diagnosis (*p* < 0.001) (Figure 3). Deaths occurring beyond 1 year after diagnosis were primarily observed among children with metabolic disease or drug‐induced cardiotoxicity. Cox regression analysis using children aged < 3 months as the reference group is shown in Table 5. Compared with the reference category, children aged > 10–18 years had a significantly higher risk of death (HR 1.92, 95% CI 1.24–2.97; *p* = 0.004), while the other age groups did not differ significantly.

**FIGURE 3:**
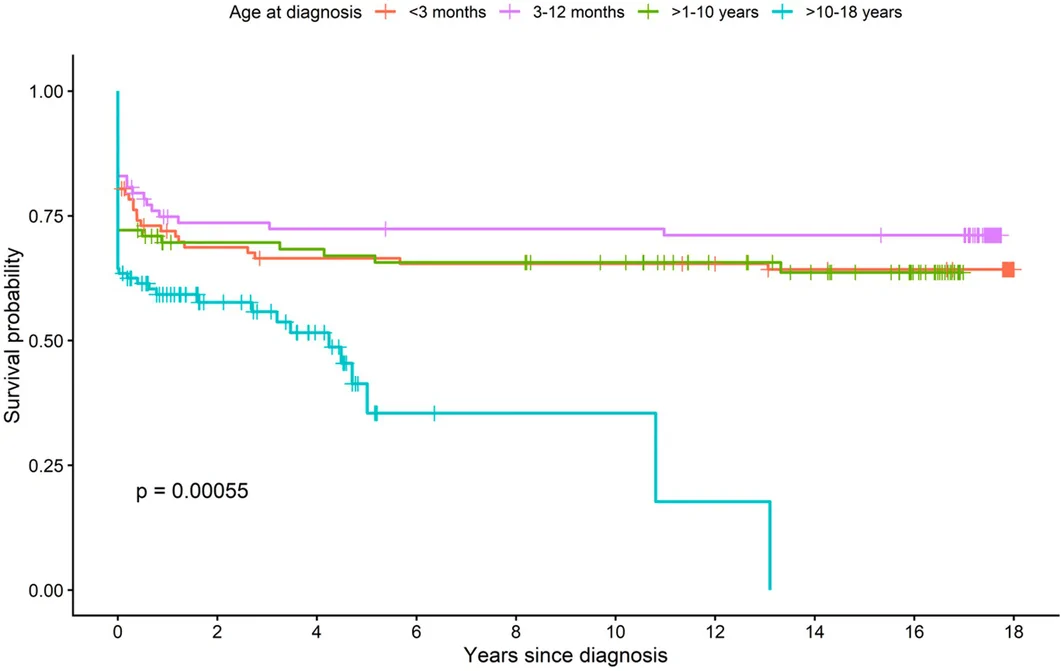
Kaplan–Meier survival curve by age groups at diagnosis. Patients with missing data at follow‐up (*n* = 16) and children with post‐mortem‐diagnosed DCM (*n* = 93) were not included in the calculation of outcomes.

**TABLE 5:** Hazard ratios for all‐cause mortality by age at diagnosis among children with dilated cardiomyopathy, 1991–2019.

Age at diagnosis	*N*	Deaths	Hazard ratio	95% CI	*p*
< 3 months	99	38	1.00	Reference	
3–12 months	88	26	0.78	0.47 to 1.31	0.346
> 1–10 years	83	32	1.06	0.65 to 1.74	0.808
> 10–18 years	107	59	1.92	1.24 to 2.97	0.004

*Note:* HRs were estimated using Cox proportional hazards regression, with children diagnosed before 3 months of age used as the reference group. HR > 1 indicates a higher hazard of all‐cause mortality compared with the reference group.

Abbreviations: CI, confidence interval; HR, hazard ratio.

## 4 Discussion

This study found an annual DCM incidence of 0.62/100 000 person‐years (95% CI 0.56–0.69). This corresponds to 12 patients with newly diagnosed DCM annually in the population of 1.9 million 0–18‐year‐olds in Sweden.

This incidence, particularly among young children, is marginally higher than that reported in earlier studies [20], which may be attributed to the inclusion of post‐mortem data and improvements in reporting practices.

The incidence is also higher than the annual incidences of childhood DCM of 0.57 and 0.58/100 000 person‐years, respectively, reported in one North American study^6^ [21], and the National Australian Childhood Cardiomyopathy Study [8]. A Finnish population‐based study from 1980 to 1991 showed an annual incidence of 0.34/100 000 person‐years for IDCM [22, 23].

Consistent with previous reports [6, 8, 21], the incidence was numerically higher among males than females in our study; however, this difference was not statistically significant. The reason for this difference remains unclear. In most cases, DCM was idiopathic, with an unknown cause of disease, making speculations about sex differences challenging. Furthermore, the difference in our cohort was modest, with 54% of all patients being male.

The clinical manifestation of DCM can be non‐specific, frequently resulting in delayed diagnosis, particularly in young children. Children adapt to their reduced exercise capacity, so the disease may be advanced by the time symptoms become apparent. Viral myocarditis may be a cause of childhood cardiomyopathy, but what percentage develops post‐viral DCM is unknown [6]. We found evidence of familial or genetic causes of cardiomyopathy in 12% of cases. This agrees with other reports of familial or genetic cardiomyopathy in children, where some suggest that up to 50% of cases may have familial or genetic causes [24]. We can only speculate on the extent of the causal contribution of genetic and environmental factors, but similarities in incidence rates across countries suggest that environmental factors play a minor role.

Some studies report better recovery in younger children [25, 26, 27], in agreement with our cohort, where children diagnosed at ≤ 10 years had better recovery and survival than older children. Most children with IDCM and post‐viral DCM were diagnosed before age 10, with favourable outcomes over time. The recovery of left ventricular function and dimensions in childhood DCM was notably high and has the potential to be sustained over time.

One‐fourth of our cases were diagnosed at post‐mortem. We included them in the incidence calculation, but we retrieved no post‐mortem reports. As in previous studies [6, 8], we did not include post‐mortem cases in our outcome calculations.

### 4.1 Implications for Clinical Practice

The outcomes of childhood DCM vary from complete recovery to death [28]. Predictive factors remain challenging to evaluate, despite recent advancements in identifying patients with the poorest prognosis [29].

In our cohort, we noticed higher mortality among patients older than 10 years at diagnosis. These results underscore the critical role of diagnostic age in predicting patient outcome, highlighting that older patients face a significantly higher death risk—a crucial insight for tailoring age‐appropriate medical interventions and improving patient management strategies.

Similar to other studies [9] with gradually improved survival over time [6, 21], we report high mortality during the first year post‐diagnosis.

The transplant‐free survival observed in our cohort appears to be consistent with findings reported in other studies. The Finnish study by Arola et al., performed when transplantation was unavailable in the Nordic countries, reported survival rates of 65%, 56% and 51%, respectively, at 1, 3 and 5 years post‐diagnosis [23]. In North America and Australia, 1‐year survival rates of 69%–72% and 5‐year survival rates of 54%–63% have been reported [6, 21].

In our cohort, the combined mortality and transplant rate among children with clinical diagnosis and available follow‐up was 49% over the study period; while more than two‐thirds of the living, non‐transplanted children with DCM regained normal heart function. This finding contrasts with findings in adults and has implications for the evaluation of different treatment protocols and indications for transplantation [30]. Indeed, only 30% of living, non‐transplanted children had persistent reduced cardiac function at the latest follow‐up, making either death/transplantation or recovery the two most likely outcomes of childhood DCM. These outcomes are at the very endpoints of the outcome spectrum, which makes the selection of treatment, especially transplantation, challenging.

### 4.2 Strengths and Limitations

This is the first population‐based national study focusing on 0–18‐year‐olds with DCM in Sweden. Identification and enrolment of cases by one paediatric cardiologist allowed a uniform and meticulous review of all patients. All seven university hospitals and central hospitals in Sweden have collaborated with this study, enabling a rich description of childhood DCM. Databases using Swedish personal identity numbers to monitor the health care of all residents were important tools. Moreover, we used the Swedish national CDR with high coverage of all deaths, including unexpected sudden deaths occurring outside hospitals.

The study provides knowledge about the occurrence and underlying pathogenic mechanisms of paediatric DCM and gives important insights into the disease. The results contribute to a better understanding of the short‐ and long‐term outcomes of childhood DCM. The information can be used to better plan the utilisation of health care resources.

However, there are limitations to consider. Only severe cases requiring hospitalisation are routinely reported to the National Board of Health and Welfare, not asymptomatic ones. One‐fourth of the patients were diagnosed at post‐mortem, but no post‐mortem reports were retrieved. Our study is a retrospective cohort analysis, and consequently, we cannot confirm that all cases were included, although we designed our study to include all possible cases. Therefore, the incidence estimates should be interpreted with some caution. However, in the setting studied, the incidence assessment is unlikely to have been influenced by selection bias.

## 5 Conclusions

This study indicates a slightly higher incidence of DCM in Sweden than previously reported, which may reflect improvements in detection methods or enhanced reporting practices.

Recovery of left ventricular function and dimensions in childhood DCM often occurs within the early years of the disease course and can be maintained over time. The challenge is to identify children with the poorest prognosis for inclusion on transplant lists while avoiding the transplantation of those who have potential for improvement or recovery.

The disease is rare but has substantial consequences, including high mortality and transplantation rates. Further studies are needed for better identification of prognostic determinants.

## Author Contributions

**Shalan Fadl:** writing – original draft, methodology, validation, visualization, writing – review and editing, investigation, conceptualization, project administration, formal analysis, software. **Tahir Taj:** writing – review and editing, formal analysis, data curation, visualization, software. **Stefan Särnblad:** writing – review and editing, project administration, data curation, supervision. **Jan Sunnegårdh:** conceptualization, investigation, funding acquisition, methodology, writing – review and editing, supervision, resources. **Håkan Wåhlander:** conceptualization, investigation, writing – review and editing, supervision. **Eva Fernlund:** writing – review and editing, investigation.

## Funding

This study was conducted with the support of Örebro University Hospital and the Örebro County Research Committee, which provided funding under grant number OLL‐524 371. Additionally, the study received financial support through the Swedish national agreement for the funding of clinical research and physician education (ALF), which provided the necessary research time.

## Conflicts of Interest

The authors declare no conflicts of interest.

## Data Availability

The datasets generated and/or analysed during the current study are not publicly available due current Swedish ethical legislation and European union GDPR act, but are available from the organisation on reasonable request, if appropriate permits are obtained from adequate authorities. Requests should be directed to: “foudatauttag@regionorebrolan.se”.
